# Metastatic Prostate Adenocarcinoma: A Rare Presentation

**DOI:** 10.7759/cureus.21292

**Published:** 2022-01-16

**Authors:** Pierre Rodriguez, Katayoun Khoshbin, Jay Vakil, Vaishali Deenadayalan, Ekrem Turk

**Affiliations:** 1 Internal Medicine, John H. Stroger, Jr. Hospital of Cook County, Chicago, USA

**Keywords:** bone metastasis, prostate specific antigen, colonoscopy, rectal nodule, prostate adenocarcinoma

## Abstract

Prostate cancer is the third most diagnosed cancer in men around the world, and it typically metastasizes to bone, lung, and liver. Gastrointestinal (GI) involvement by prostate cancer is rare, as patients tend to present with upper and lower GI bleed among other symptoms not related to prostate cancer, which commonly include lower urinary tract symptoms such as urinary frequency, dribbling of urine, or urinary retention. In cases of patients with prostate cancer and symptoms from the GI system, colonoscopy and biopsy of lesions should be performed to allow physicians to make an accurate and prompt diagnosis in patients with metastatic prostate cancer with rectal involvement. We present a case of a patient who initially complained of melena and was found to have a rectal nodule with biopsy-proven metastatic prostate cancer.

## Introduction

Prostate cancer often presents with localized disease at the time of diagnosis; however, it can also present with evidence of metastatic disease at presentation. Although bone is the most frequent location for metastatic prostate cancer, atypical metastases often present in patients delaying care and an accurate diagnosis. A rare presentation of metastatic prostate adenocarcinoma includes rectal involvement, with patients commonly presenting with upper and lower gastrointestinal (GI) symptoms such as rectal pain, hematochezia, melena, or altered bowel movements, requiring prompt evaluation with upper and lower endoscopy; however, it is not until biopsy results are reported that an accurate diagnosis can be made. We report a case of an elderly Hispanic male presenting initially with melena, who was found to have a rectal nodule, with biopsy showing prostate adenocarcinoma.

## Case presentation

**Figure 1:**
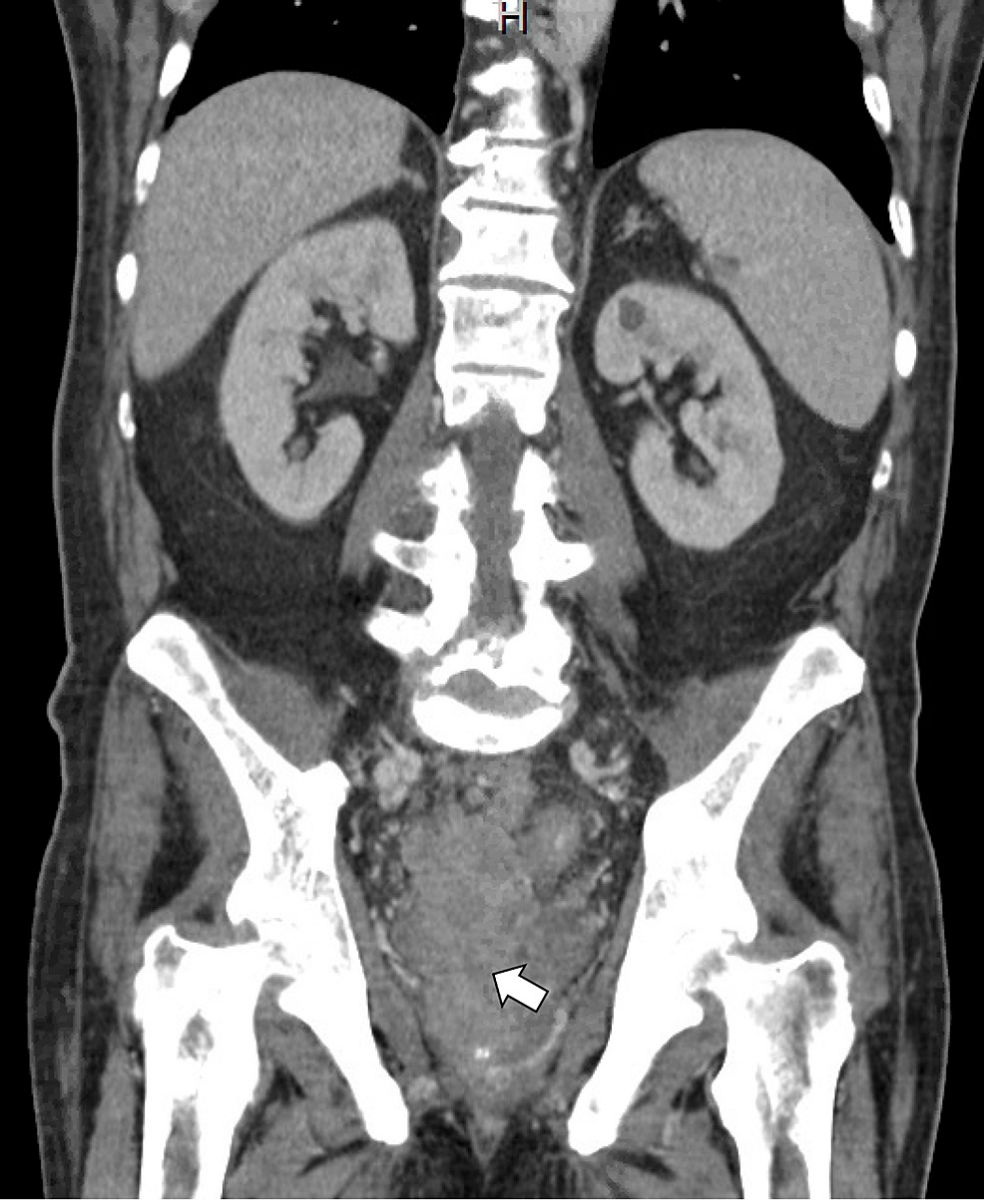
CT scan (coronal plane) of the abdomen and pelvis White arrowhead shows tumor arising from the prostate invading the pelvis CT, computed tomography

**Figure 2:**
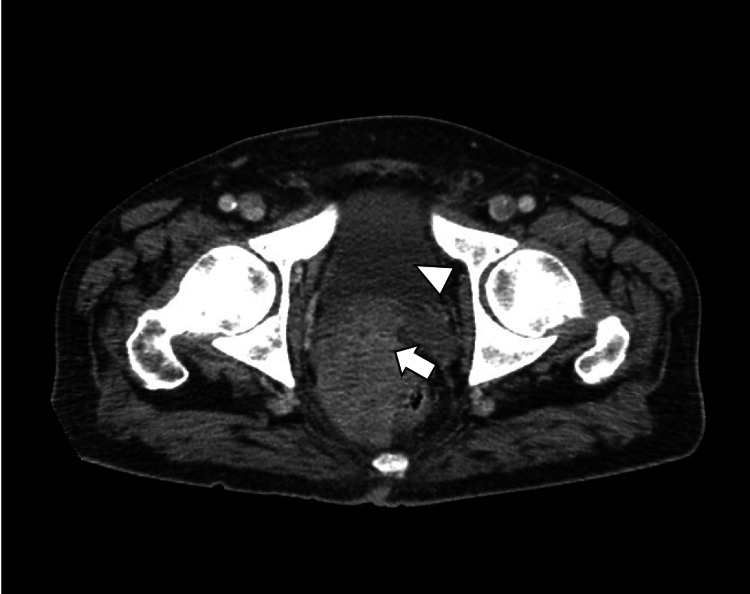
CT scan (transverse view) of the abdomen and pelvis White arrowhead shows urinary bladder, and white arrow shows tumor arising from the prostate compressing the base of the urinary bladder and extending posteriorly towards the rectum CT, computed tomography

**Figure 3:**
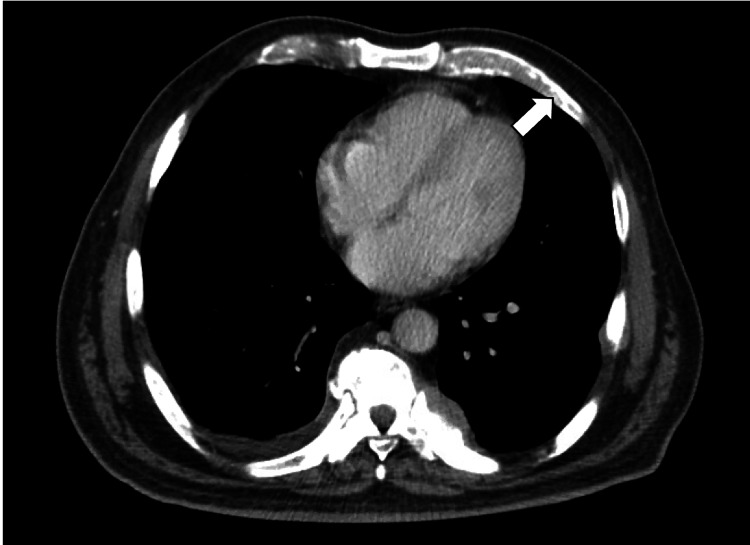
CT of the chest White arrow shows extensive mixed sclerotic/lytic metastasis throughout the ribs CT, computed tomography

**Figure 4:**
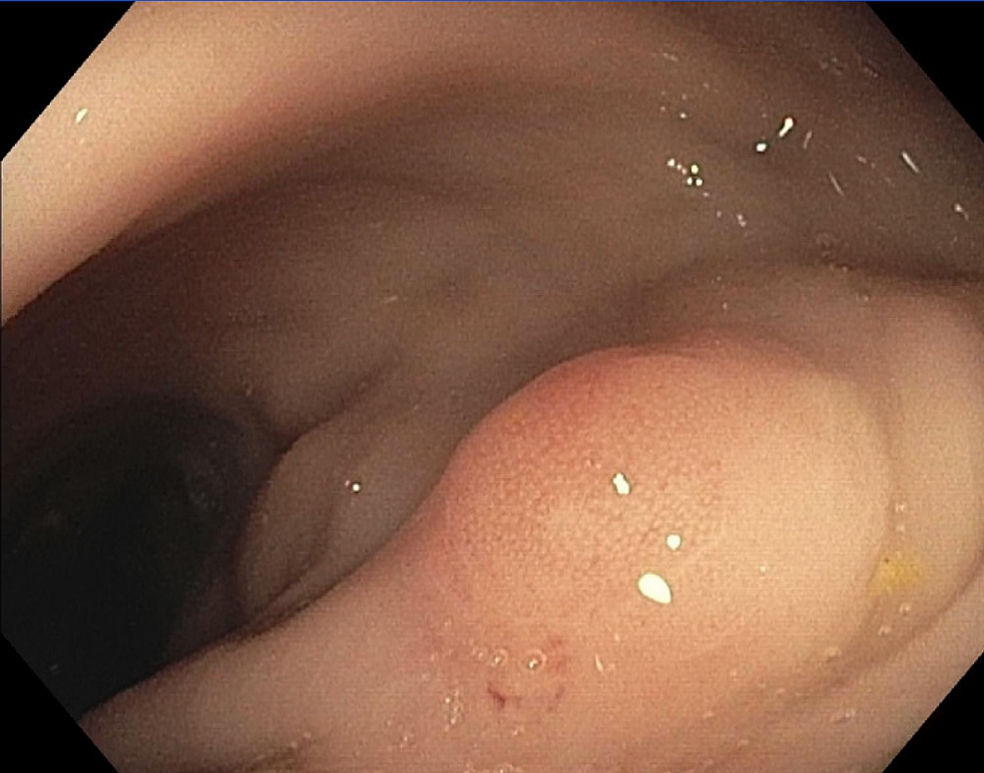
Rectal nodule (10 cm from the anal verge)

**Figure 5:**
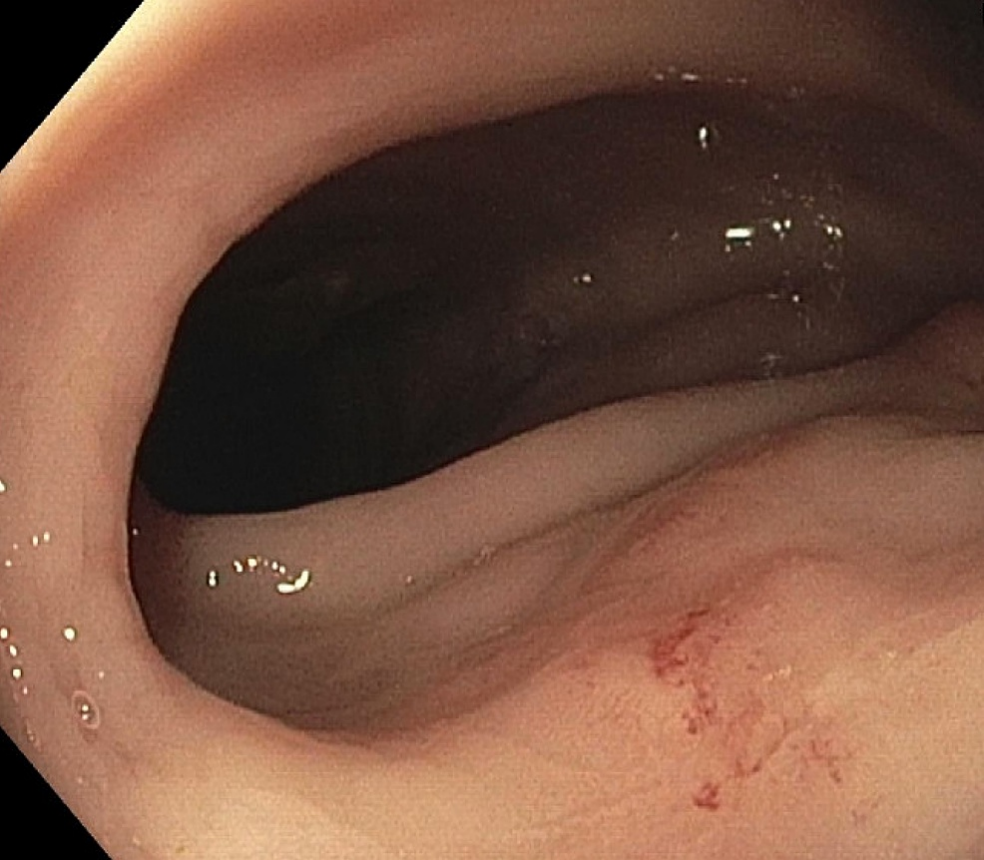
Erythema surrounding the rectal nodule

A 67-year-old Hispanic male with a past medical history of type 2 diabetes, hypertension, and hyperlipidemia presented to the ER with complaints of generalized weakness, fatigue, and back pain for six months. The patient initially noticed increasing and worsening urinary hesitancy, urinary frequency, urine dribbling, and pressure in the lower abdomen, at the level of the hypogastrium. Symptoms were followed by four months of worsening bilateral sharp and shooting back pain radiating to bilateral lower extremities without weakness or sensory deficits, with associated intermittent constipation requiring stool softeners. He also endorsed episodes of dark tarry stools concerning for melena with associated weight loss of approximately 40 pounds in six months, with decreased appetite but no dysphagia, nausea, or vomiting. Computed tomography (CT) scan of the abdomen showed a large heterogeneously enhancing infiltrating mass within the pelvis (Figure 1), likely arising from the prostate and extending posteriorly and superiorly likely involving the rectum and base of urinary bladder (Figure 2) and extensive mixed sclerotic/lytic metastasis throughout the skeleton and spine (Figure 3). Magnetic resonance imaging (MRI) of the lumbar spine showed extensive osseous disease but no pathological fracture or spinal canal compromise. Two weeks prior to this hospitalization, esophagogastroduodenoscopy (EGD) was performed at an outside hospital, which showed positivity for *Heliobacter pylori* that the patient completed antibiotics for. Due to weight loss and persistent concern for upper GI bleed and laboratory consistent with iron deficiency anemia, colonoscopy was also performed, which showed normal-appearing terminal ileum, superficial healing ulcer measuring around 12 to 15 mm in size in the hepatic flexure without evidence of colitis, and a firm, sub-epithelial nodule measuring around 7 mm in the rectum, around 10 cm from the anal verge (Figure 4) with patchy erythematous mucosa with diminutive erosions seen adjacent to the rectal nodule (Figure 5) and an area of extrinsic compression seen in the rectum. Pathology later showed prostate adenocarcinoma in the rectal nodule biopsy with prostate-specific antigen (PSA) stain positive and negative for CDX2 and synaptophysin, respectively. Prostate biopsy was performed as well, which showed adenocarcinoma, with a Gleason score of 5+4 = 9. Skeletal bone survey showed extensive mixed sclerotic metastasis throughout the skeleton and spine. The patient underwent prophylactic intramedullary (IM) nailing of the right proximal femur followed by radiation therapy to the right femur and external beam radiotherapy (EBRT) to T7-S2.

## Discussion

Prostate cancer is the third most commonly diagnosed malignancy in the world, only preceded by lung and colorectal cancers. According to the SEER (Surveillance, Epidemiology and End Results) model, Americans have a one in nine probability of developing invasive prostate cancer in their lifetime. In its early stages, prostate cancer is commonly asymptomatic. Symptomatic disease usually implies worse disease, with curative treatment becoming difficult. At the time of diagnosis, 78% of patients have localized cancer, 12% have regional lymph node involvement, and 6% have distant metastases [1]. The implementation of widespread PSA screening in the United States in the late 1980s significantly increased the detection of prostate cancer. Detection of the disease in its early stages allowed for greater options for treatment. As a result, increased screening practices with PSA testing led to a substantial measured drop in prostate cancer-specific mortality by 37% [2].

As mentioned earlier, prostate cancer is commonly asymptomatic. Symptoms occur usually due to expansion of the mass causing urethral compression. The most common complaints men have are usually urinary retention, urinary frequency, hesitancy, and nocturia [3]. These symptoms commonly overlap with those of benign prostatic hyperplasia (BPH), and it may be difficult to differentiate between the two. Our patient also complained of obstructive symptoms, such as those mentioned, but he also had a history of BPH and prior elevated PSA that could explain them.

This case highlights two main points to consider. The first point is that masses that appear in the rectal wall may not always be rectal cancers. It is important to consider prostate adenocarcinoma in the differential diagnosis in men who present with such masses/nodules. This holds especially true when patients have accompanying urinary symptoms or may have a previous diagnosis of BPH. The next point this case presents is the role of PSA in the diagnosis of prostate cancers. PSA is the most common oncological marker used for the screening of prostate cancer. Unfortunately, BPH, as well as prostatitis, can result in high levels of PSA, which decreases its specificity as a cancer marker. This commonly leads to unneeded prostate biopsies and added costs for those who may only have BPH [4]. For this reason, providers should keep closer attention to PSA levels as well as the symptoms of their patients with BPH. To add, PSA as a screening marker has been controversial for some in the past. Many studies are evaluating alternative markers that may prove beneficial for the diagnosis of prostate cancer. For now, the U.S. Preventive Services Task Force (USPSTF) recommends that screening may be useful among men between 55 and 69 years of age, but the decision to test should be made on a case-by-case basis after the patient understands the risks and benefits of screening.

In addition to urinary symptoms, our patient complained of chest, back, and bilateral lower extremity pain. He was found to have extensive metastases throughout the skeleton and spine, likely explaining his symptoms. Patients with advanced stage prostate cancer often develop metastases to bone. When bone involvement is present, patients may experience skeletal/bone pains and may commonly present with fractures. It is thought that increased levels of PTHrP induce bone resorption and allow for the creation of a microenvironment hospitable for tumor cells [5].

Uncommon in the presentation of prostate cancer, though, is the presence of melena. Our patient complained of constipation, pain passing stools, and black tarry stools. These symptoms, consistent with GI bleeding, prompted further evaluation via colonoscopy. In his colonoscopy, a rectal nodule was discovered, which stained positive for PSA and allowed for the diagnosis of prostate adenocarcinoma. Prostate cancers presenting as rectal masses are extremely uncommon. In a 2017 study evaluating the histology of rectal masses, it was found that of the 9,504 patients evaluated, nine had prostate cancers clinically misdiagnosed at rectal cancers [6]. Prostate cancer normally metastasizes to the bones, lungs, and liver. Denonvilliers' fascia separates the rectum from the prostate, and it is the main reason why prostate cancer rarely invades the rectum; however, autopsy series showed that 0.56% to 11.5% of all cases of prostate carcinoma can present as a rectal mass [7] . Rectal involvement can happen through direct invasion through the fascia, shared lymph nodes, and seeding through needle biopsy [8]. Treatment for metastatic prostate cancer includes EBRT, hormone therapy including ADT (androgen deprivation therapy), chemotherapy, and surgery including radical prostatectomy [9]. Our patient received EBRT to T7-S2 and right femur followed by prophylactic IM nailing of the right femur, followed by ADT with degarelix and goserelin every three months and chemotherapy with docetaxel.

## Conclusions

Metastatic prostate cancer presenting with rectal involvement is rare and has been reported in the literature. Patients often present with symptoms concerning for upper and lower GI bleeding requiring upper and lower endoscopy. Our patient had a rectal nodule that was found to have positive stains for prostate cancer, and an accurate diagnosis of prostate adenocarcinoma was made upon results. This case report highlights the importance of keeping a broad differential diagnosis in patients who are found to have rectal nodule to differentiate between primary GI malignancy and metastatic prostate cancer in order to treat patients adequately based on pathology results.
